# Supplementary material for: Approaching isotropic charge transport of n-type organic semiconductors with bulky substituents
Source: Commun Chem. 2021 Nov 11;4:155. doi: 10.1038/s42004-021-00583-2 (PMC9814529; doi:10.1038/s42004-021-00583-2)
Supplement: Supplementary file 1 — Supplementary Information [file 42004_2021_583_MOESM1_ESM.pdf]

Supplementary Information for

Approaching isotropic charge transport of n-type organic semiconductors with bulky substituents

Craig P. Yu[1], Naoya Kojima[2], Shohei Kumagai[1], Tadanori Kurosawa[1], Hiroyuki Ishii[3], Go Watanabe[4], Jun Takeya[1,2,5,6], and Toshihiro Okamoto[1,2,5,7,8*]

[1]Material Innovation Research Center (MIRC) and Department of Advanced Materials Science, School of Frontier Sciences, The University of Tokyo, 5-1-5 Kashiwanoha, Kashiwa, Chiba 277-8561, Japan

[2]Department of Applied Chemistry, Faculty of Engineering, The University of Tokyo, 7-3-1 Hongo, Bunkyo-ku, Tokyo 113-0033, Japan

[3]Department of Applied Physics, Faculty of Pure and Applied Sciences, University of Tsukuba, 1-1-1 Tennodai, Tsukuba, Ibaraki 305-8573, Japan

[4]Department of Physics, School of Science, Kitasato University, 1-15-1 Kitasato, Minami-ku, Sagamihara, Kanagawa 252-0373, Japan.

[5]National Institute of Advanced Industrial Science and Technology (AIST)-University of Tokyo Advanced Operando-Measurement Technology Open Innovation Laboratory (OPERANDO-OIL), AIST, 5-1-5 Kashiwanoha, Kashiwa, Chiba 277-8561, Japan

[6]International Center for Materials Nanoarchitectonics (MANA), National Institute for Materials Science (NIMS), 1-1 Namiki, Tsukuba 205-0044, Japan

[7]PRESTO, JST, 4-1-8 Honcho, Kawaguchi, Saitama 332-0012, Japan

[8]CREST, JST, 4-1-8 Honcho, Kawaguchi, Saitama 332-0012, Japan

*Corresponding Author:

Toshihiro Okamoto, tokamoto@k.u-tokyo.ac.jp

1. ^{1}H NMR Spectra 3
2. Thermal Properties 6
3. Electrochemistry 6
4. Solubility Tests 7
5. X-Ray Crystallography 8
6. Theoretical Calculations 11
7. Single-Crystalline Thin-Film OFET Performances 14
8. Thin-Film Surface Morphology 15
9. Polycrystalline Thin-Film Assemblies of Ph–BQQDI and Cy_6–BQQDI 17
10. Polycrystalline Thin-Film OFET Performances 19
11. 2D LUMO Bands 21
12. Anisotropy of Electron Mobility 22
13. Supplementary References 24

1. ^{1}H NMR Spectra

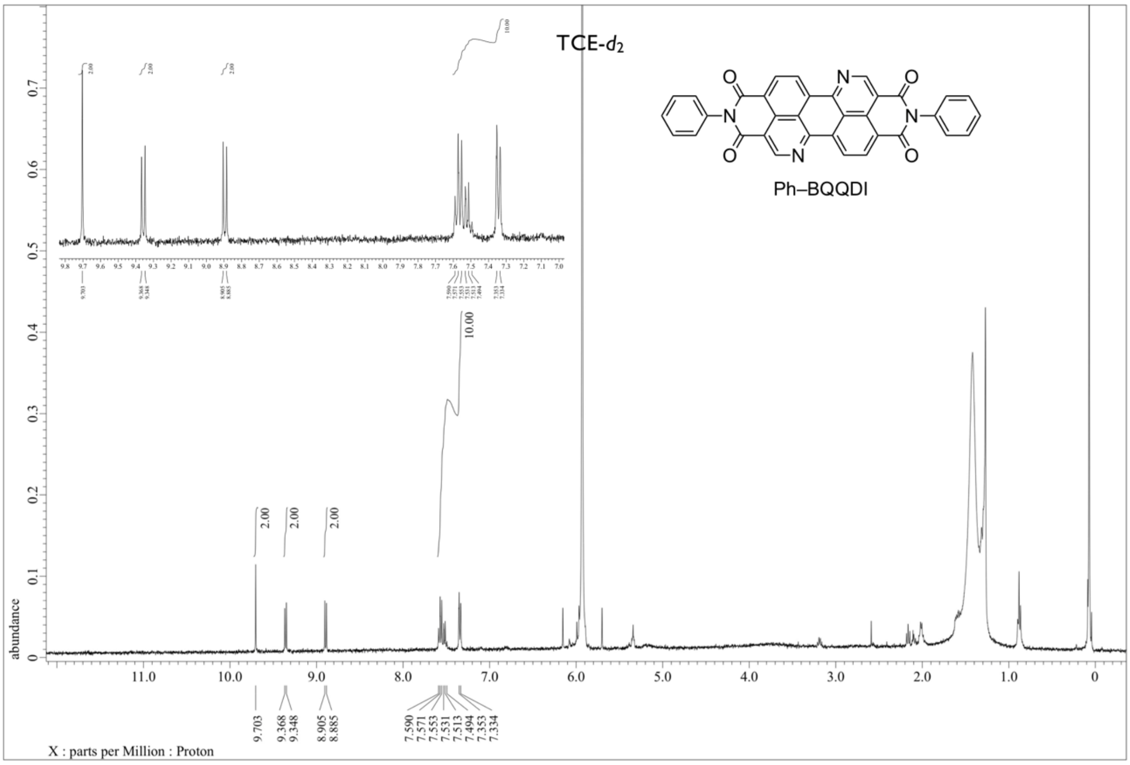

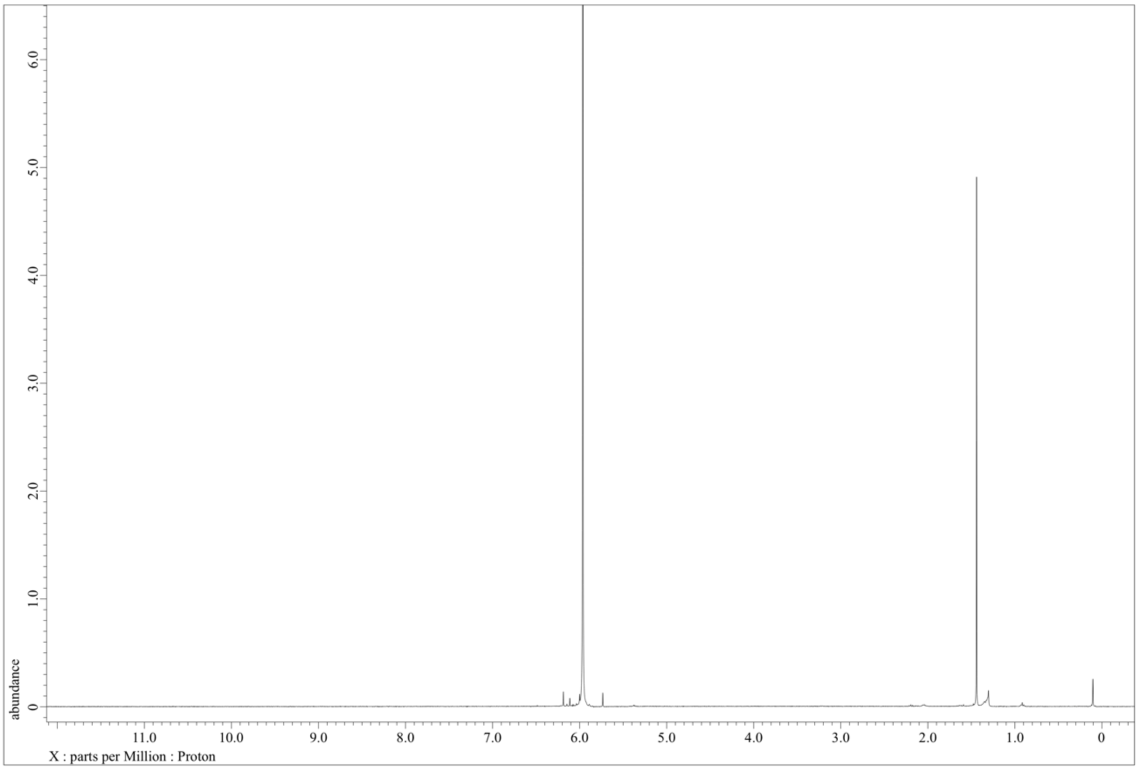

Supplementary Fig. 1 The ^{1}H NMR spectrum of Ph–BQQDI in TCE-d_2 at 100 ˚C (top), blank TCE-d_2 spectrum at 100 ˚C (bottom).

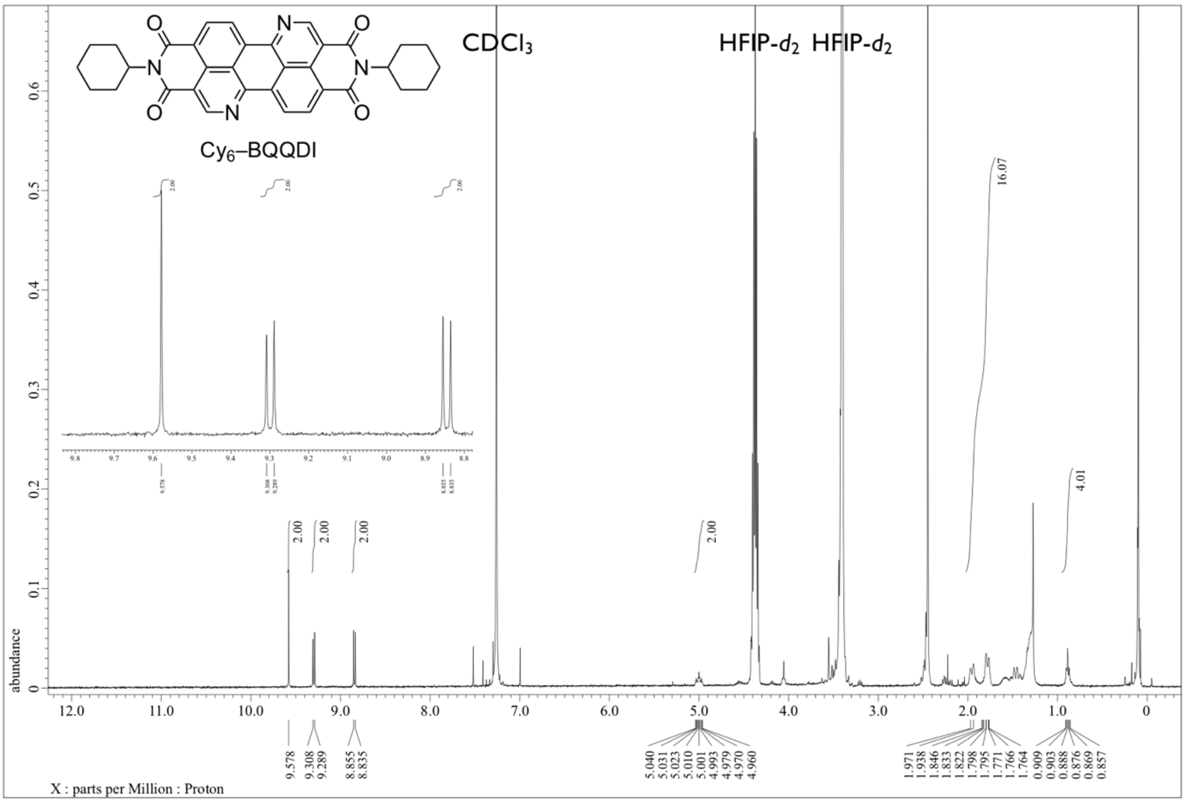

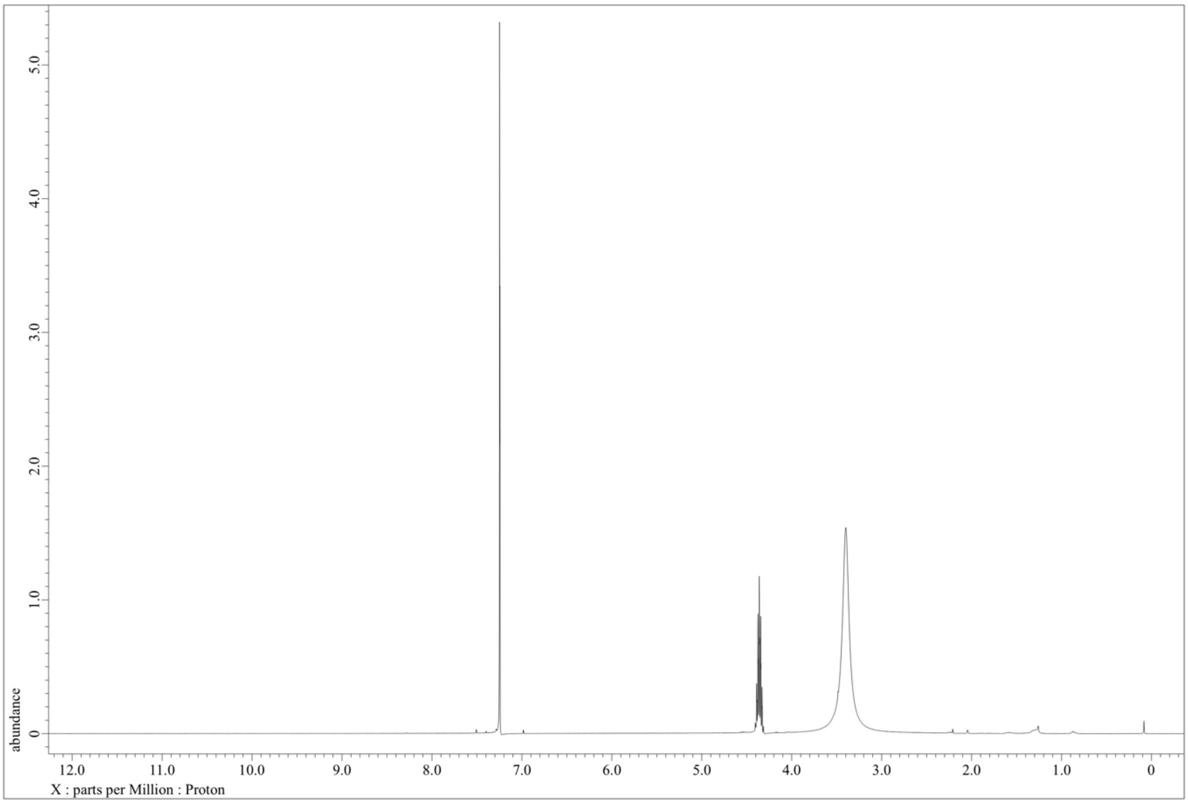

Supplementary Fig. 2 The ^{1}H NMR spectrum of Cy$_6$–BQQDI in $CDCl_3$/HFIP-d$_2$ at room temperature (top), blank $CDCl_3$/HFIP-d$_2$ spectrum at room temperature (bottom).

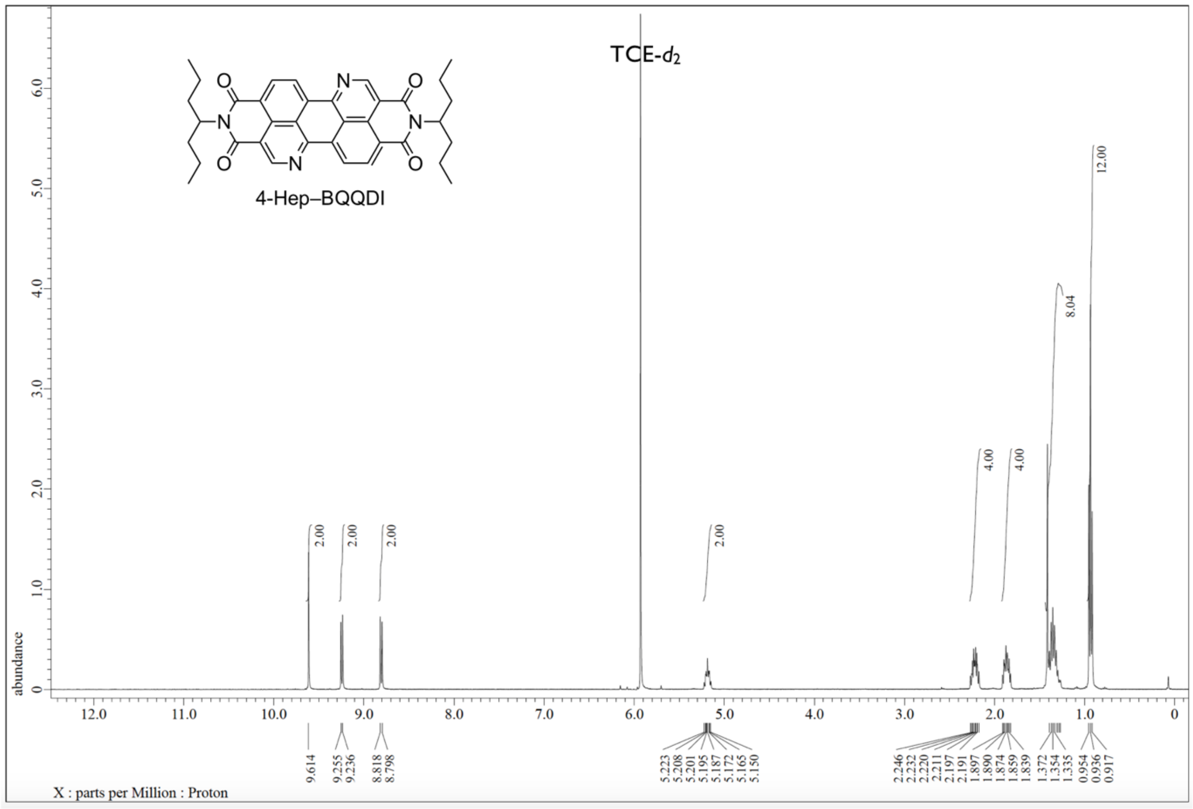

Supplementary Fig. 3 The ^{1}H NMR spectrum of 4-Hep–BQQDI in TCE-d_2 at 100 ˚C.

2. Thermal Properties

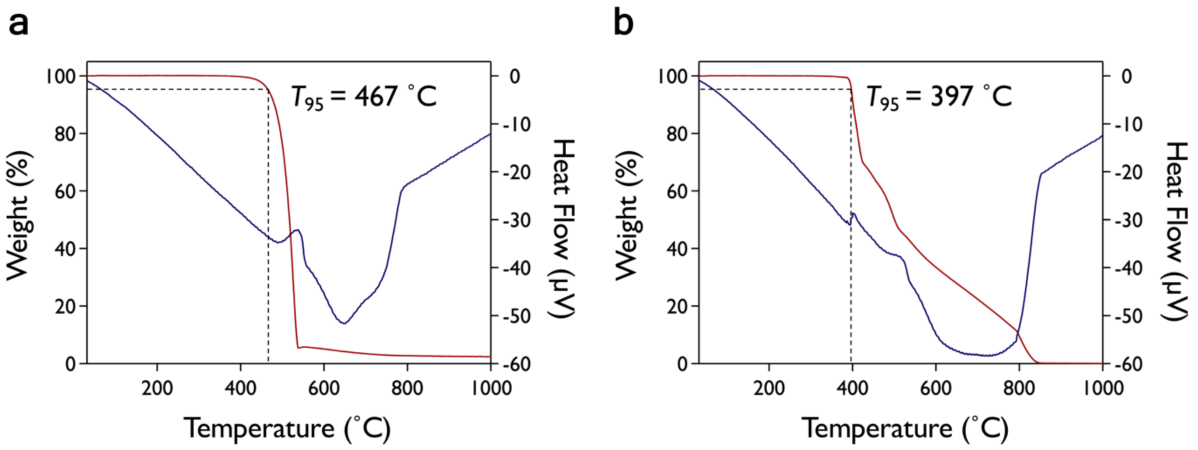

Supplementary Fig. 4 TG-DTA curves of **a** Ph–BQQDI, and **b** Cy_6–BQQDI.

3. Electrochemistry

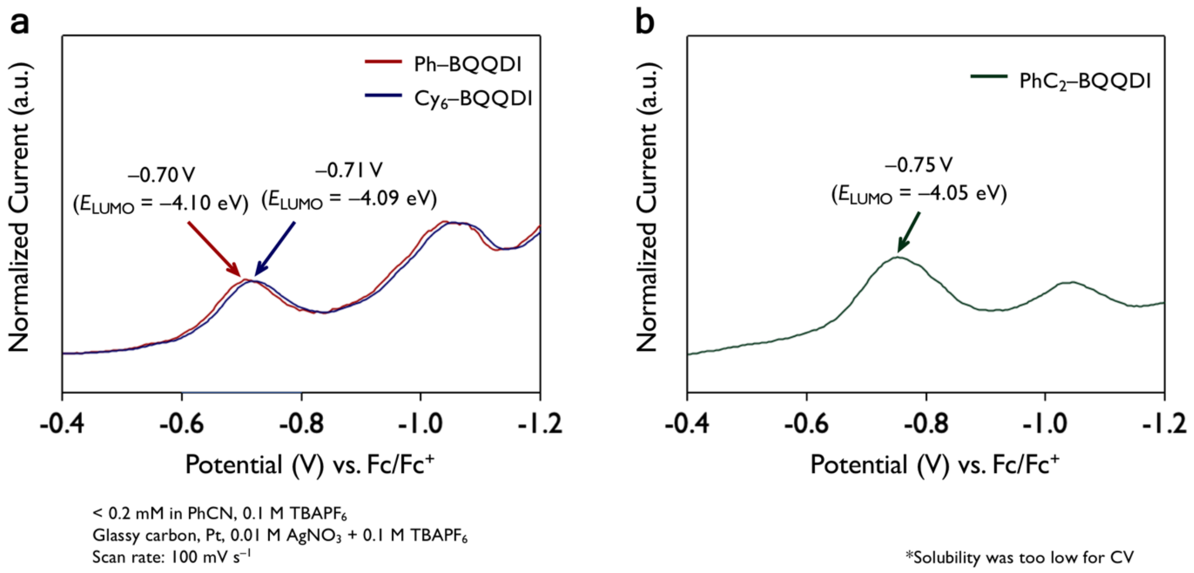

Supplementary Fig. 5 Differential pulse voltammetry and their corresponding LUMO energy levels of **a** Ph– and Cy_6–BQQDI, and **b** PhC_2–BQQDI as a reference.

4. Solubility Tests

Supplementary Table 1 Solubility of R–BQQDI.

Compound	Solubility (wt%)
PhC_2–BQQDI	0.038[1]
Cy_6–BQQDI	0.0042
Ph–BQQDI	< 0.001

5. X-Ray Crystallography

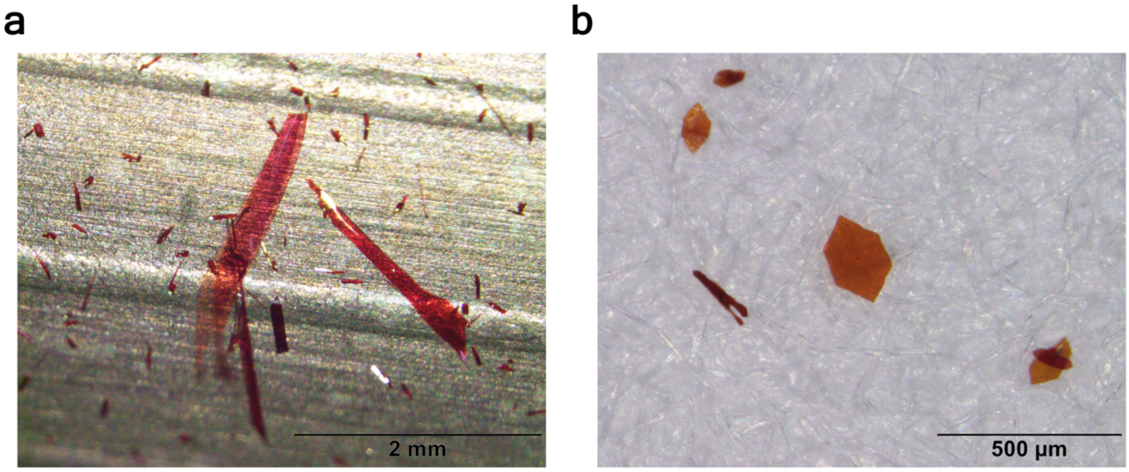

Supplementary Fig. 6 Microscopic images of single-crystals of **a** Ph–BQQDI, and **b** Cy_6–BQQDI.

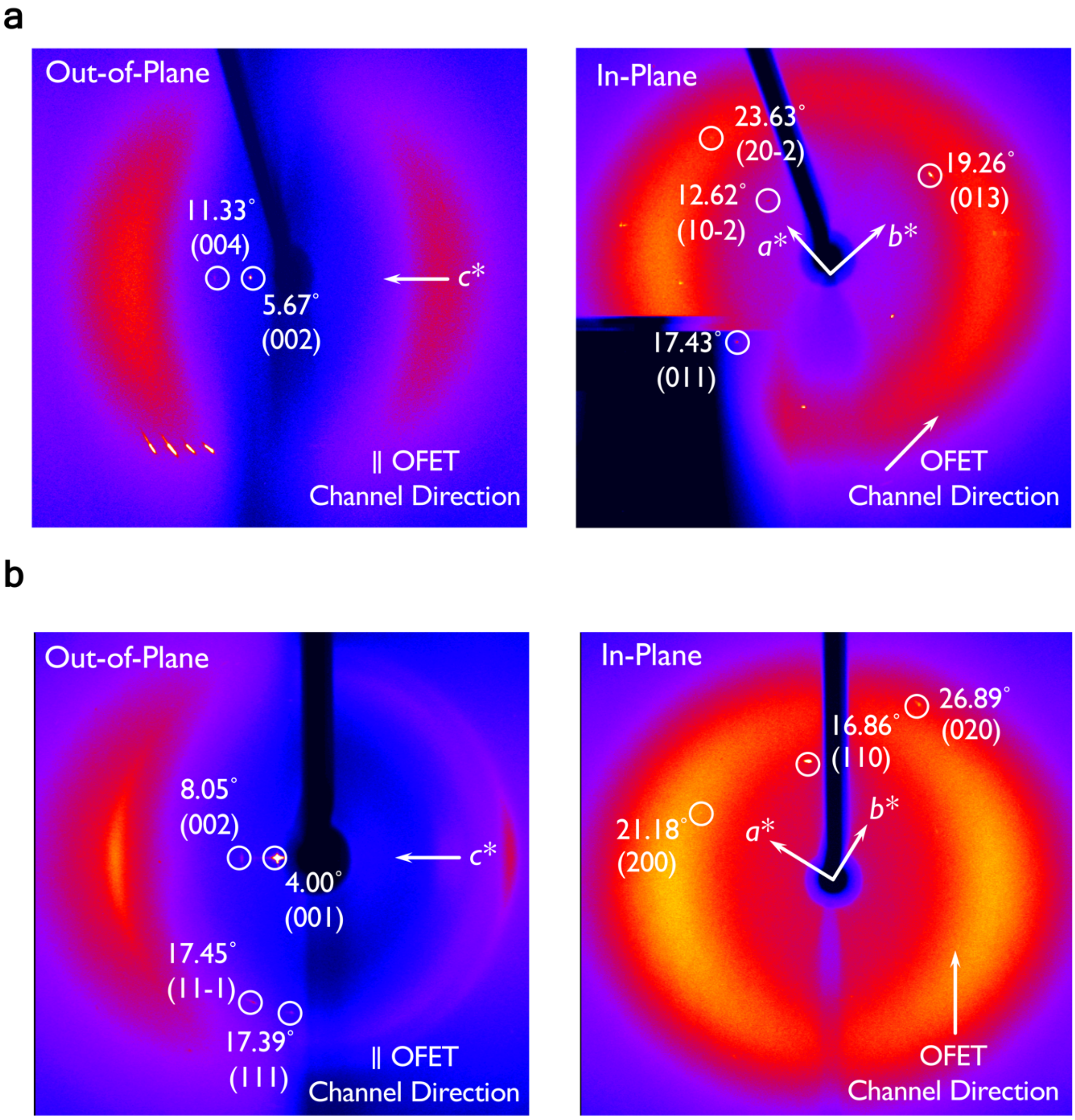

Supplementary Fig. 7 Thin-film transmission X-ray diffraction images of **a** Ph–BQQDI and **b** Cy_6–BQQDI.

Supplementary Table 2 Single-crystal data of Ph–BQQDI and Cy_6–BQQDI.

Material	Ph–BQQDI	Cy_6–BQQDI
Formula	$C_{34}H_{16}N_4O_4$	$C_{34}H_{28}N_4O_4$
Formula weight	544.52	556.60
Crystal system	monoclinic	monoclinic
Space group	$P2_1/c$	$C2/m$
a / Å	7.6097(4)	8.5200(6)
b / Å	5.1587(2)	6.6538(6)
c / Å	31.1537(14)	22.3522(18)
α / °	90	90
β / °	93.645(7)	98.917(7)
γ / °	90	90
V / Å^3	1220.50(10)	1251.84(18)
Z	2	2
T / K	298	297
R_1, wR_2 [$I > 2\sigma(I)$]	0.0682, 0.2098	0.0494, 0.1425
R_1, wR_2 [all data]	0.1214, 0.2341	0.0900, 0.1711
GOF	1.135	0.962

6. Theoretical Calculations

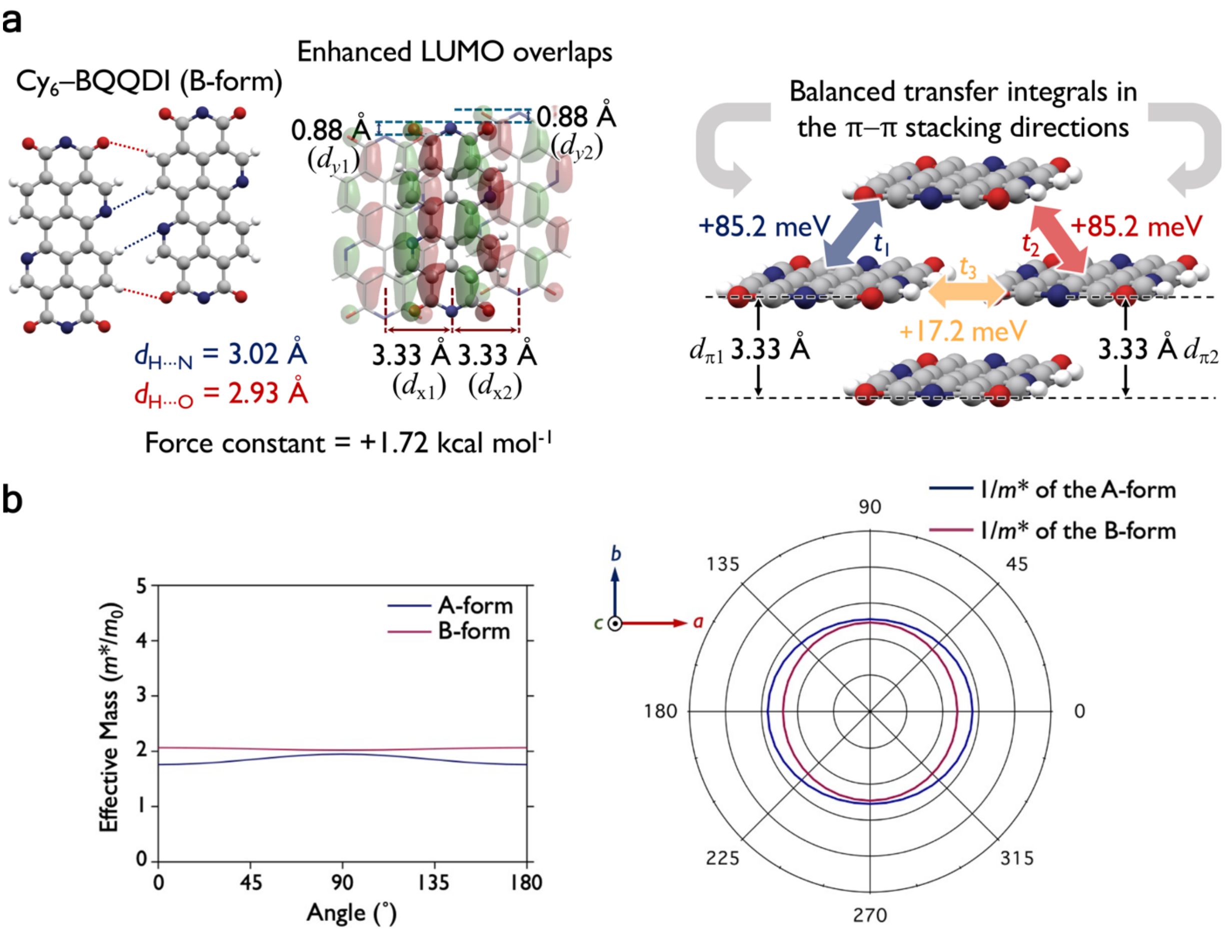

Supplementary Fig. 8 **a** Intermolecular distances, interactions and LUMO overlaps, stacking distances (between planes of atoms on the BQQ core, excluding hydrogens), and transfer integrals of Cy_6–BQQDI (B form). **b** Effective and angle-dependent inversed effective mass of Cy_6–BQQDI (A- and B-forms).

Supplementary Table 3 Number of molecules, unit-cell parameters of the initial MD cell.

Material	Ph–BQQDI	Cy_6–BQQDI (A-form)	Cy_6–BQQDI (B-form)
Number of molecules	672	576	576
T / K	298	297	297
a / nm	6.0878	5.1120	5.1120
b / nm	7.2222	5.3230	5.3230
c / nm	9.3461	13.4113	13.4113
α / °	90.00	90.00	90.00
β / °	93.64	98.92	98.92
γ / °	90.00	90.00	90.00

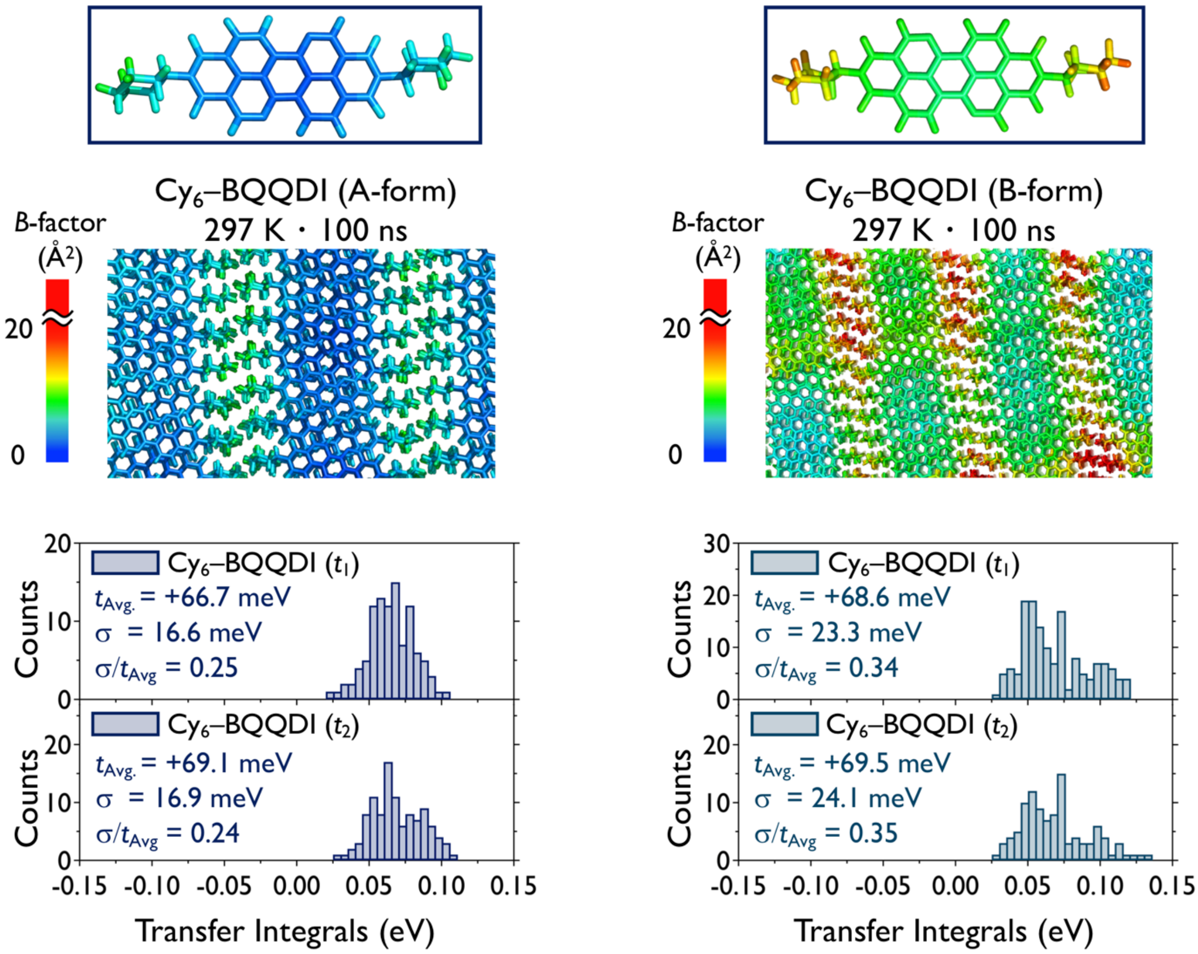

Supplementary Fig. 9 Color-coded B-factor (Å^2) distributions of Cy$_6$–BQQDI (A- and B forms) obtained from the trajectories during the last 10 ns of a 100 ns MD simulations in the NTP ensemble (the magnitude of B-factors is represented by the color-coded scale bar ranging from blue (small value) to red (large value). Variant t_1 and t_2 distributions and standard deviations (σ) calculated from more than 100 pairs of adjacent dimers revealing the magnitude of the molecular fluctuations.

7. Single-Crystalline Thin-Film OFET Performances

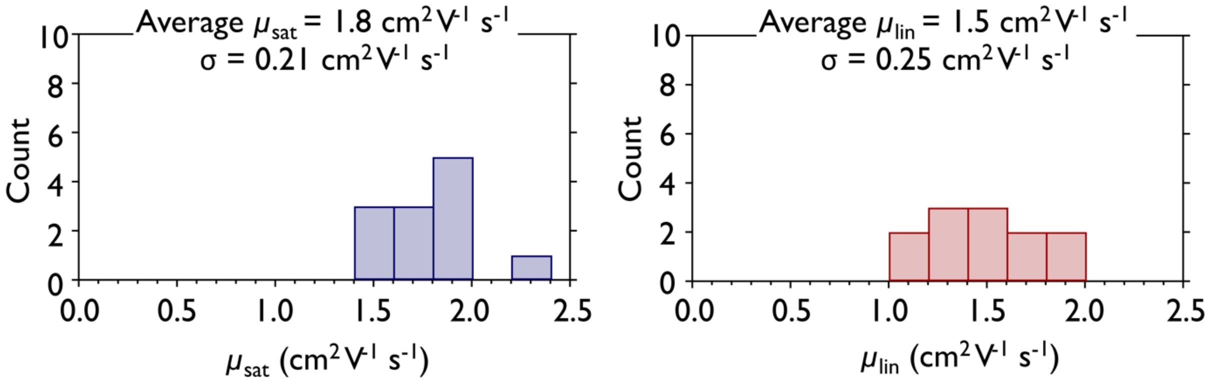

Supplementary Fig. 10 Statistical distribution of μ_{sat} and μ_{lin} of Cy_6–BQQDI-based single-crystalline OFETs.

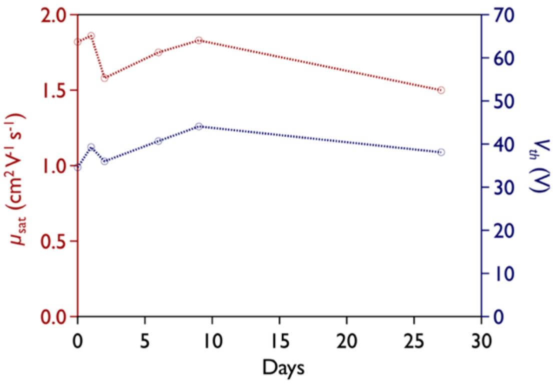

Supplementary Fig. 11 Stability of μ_{sat} and V_{th} of Cy_6–BQQDI-based single-crystalline OFETs.

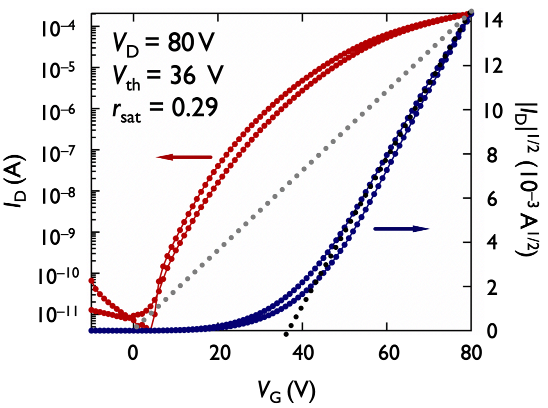

Supplementary Fig. 12 Reliability factor r_{sat} of Cy_6–BQQDI-based OFET showing the maximum μ_e, where black and gray dashed lines represent the fit to $|I_D|^{1/2}$ and the slope of an electrically ideal OFET, respectively.

8. Thin-Film Surface Morphology

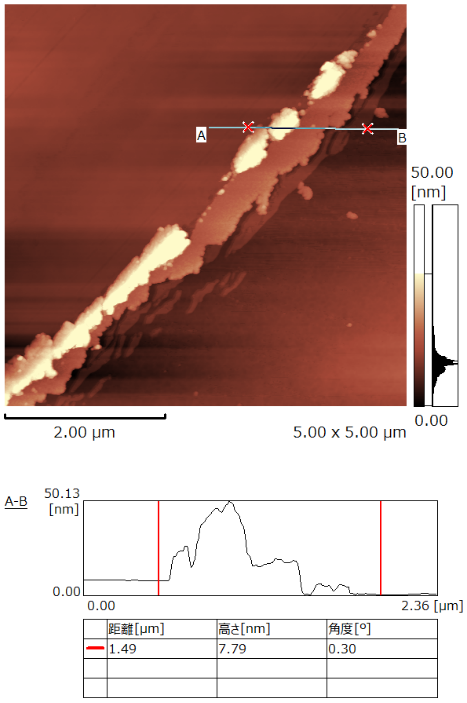

Supplementary Fig. 13 AFM images of the single-crystalline thin film of Cy$_6$–BQQDI fabricated via the edge-casting method.

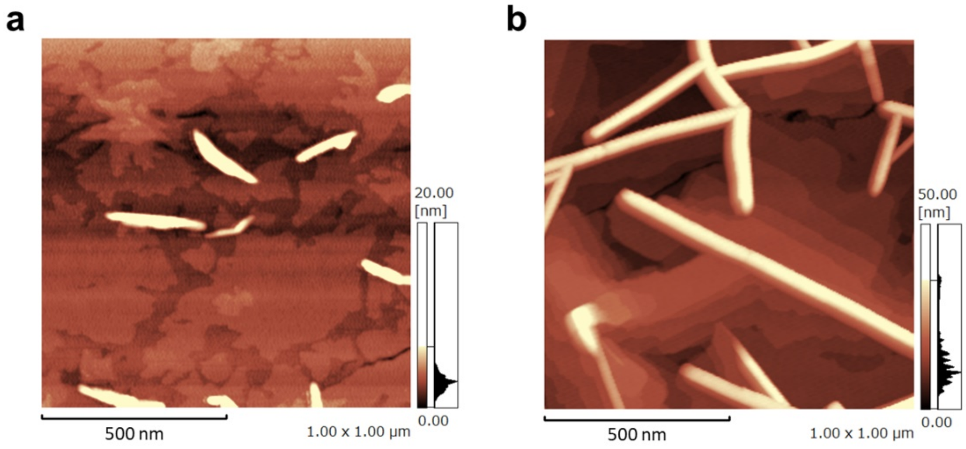

Supplementary Fig. 14 AFM images of **a** Ph–BQQDI and **b** Cy$_6$–BQQDI, on DTS with thin-film thickness of 40 nm.

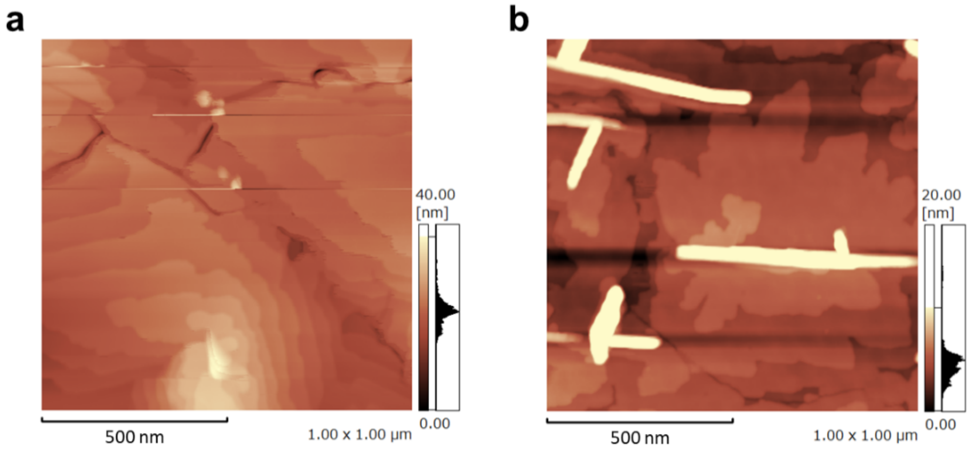

Supplementary Fig. 15 AFM images of Cy_6–BQQDI on HMDS with thicknesses of **a** 40 nm and **b** 20 nm.

9. Polycrystalline Thin-Film Assemblies of Ph–BQQDI and Cy_6–BQQDI

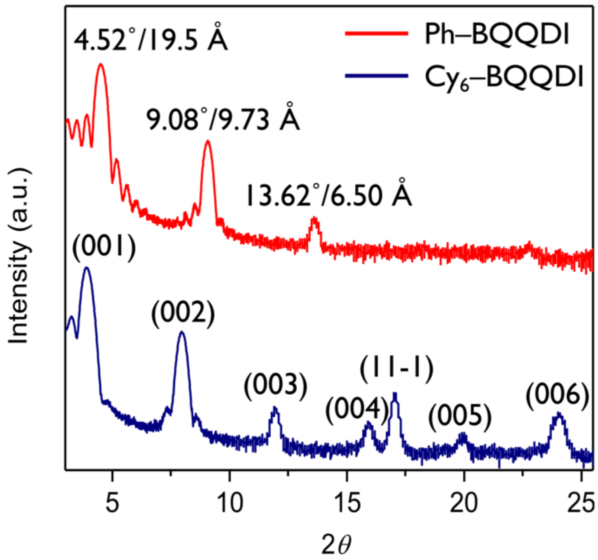

Supplementary Fig. 16 Out-of-plane polycrystalline thin-film X-ray diffractions of Ph– and Cy_6–BQQDI on DTS.

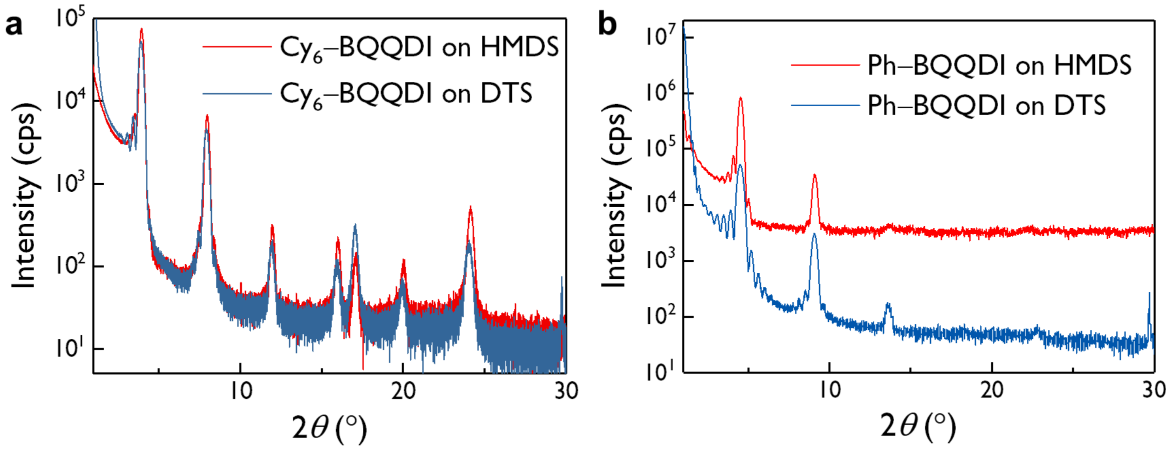

Supplementary Fig. 17 Out-of-plane polycrystalline thin-film X-ray diffractions of **a** Cy_6–BQQDI and **b** Ph–BQQDI on DTS and HDMS.

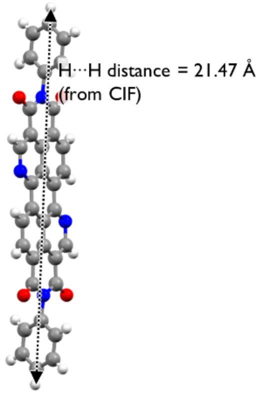

Supplementary Fig. 18 The longest intramolecular H···H distance (molecular length) in the single crystal structure.

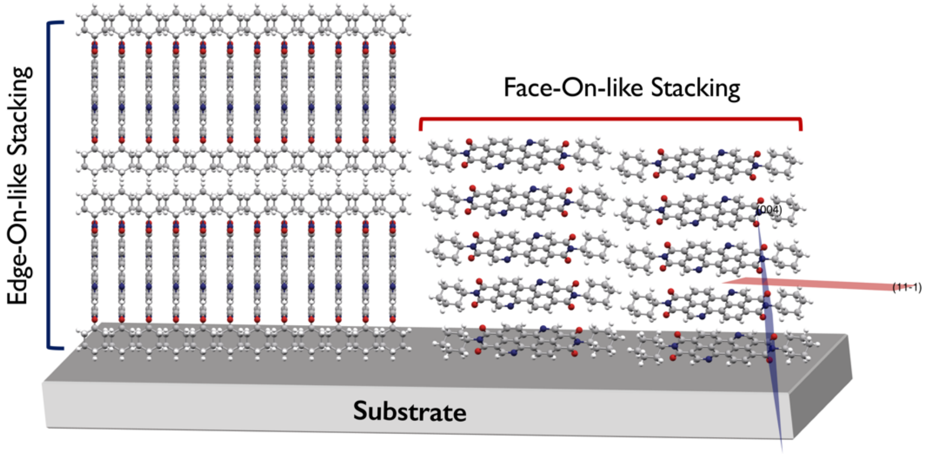

Supplementary Fig. 19 Edge-on and face-on-like stacking of polycrystalline Cy_6–BQQDI on the OFET substrate.

10. Polycrystalline Thin-Film OFET Performances

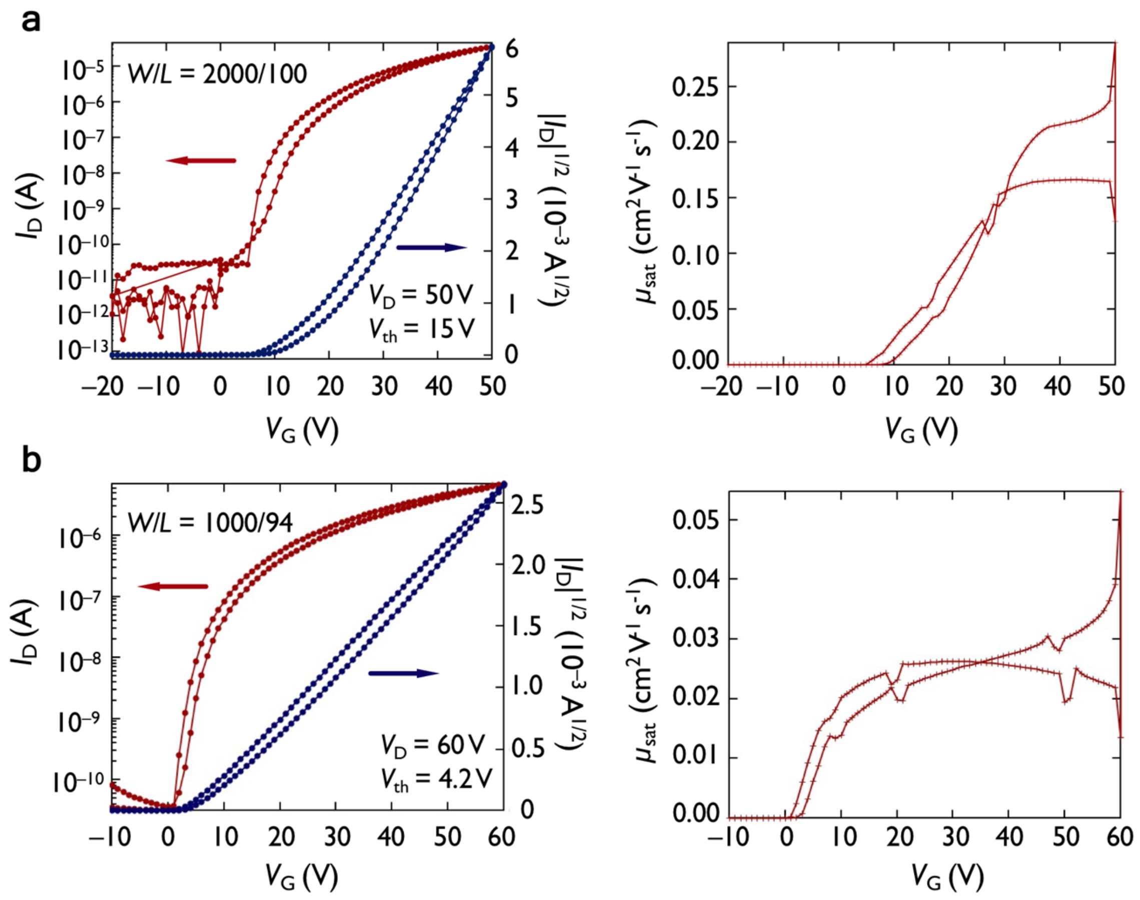

Supplementary Fig. 20 Transfer characteristics and μ_{sat} of 40 nm-thick Ph–BQQDI polycrystalline OFET on **a** DTS, and **b** HDMS.

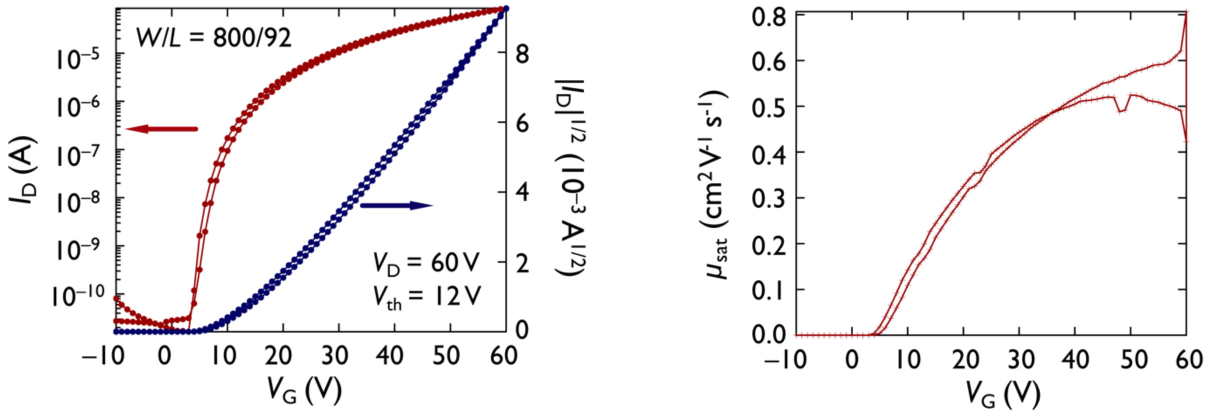

Supplementary Fig. 21 Transfer characteristics and μ_{sat} of 40 nm-thick Cy_6–BQQDI polycrystalline OFET on DTS.

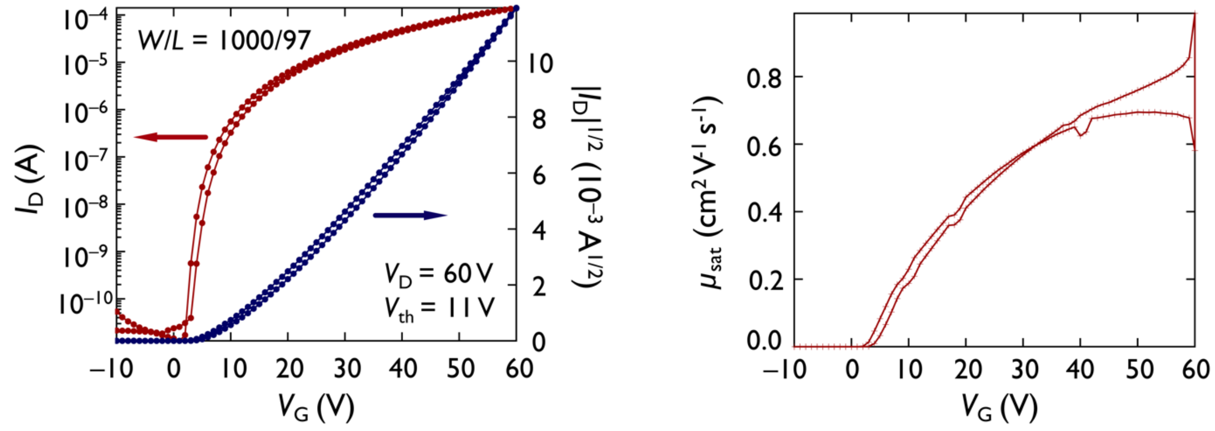

Supplementary Fig. 22 Transfer characteristics and μ_{sat} of 40 nm-thick Cy6–BQQDI polycrystalline OFET on HMDS.

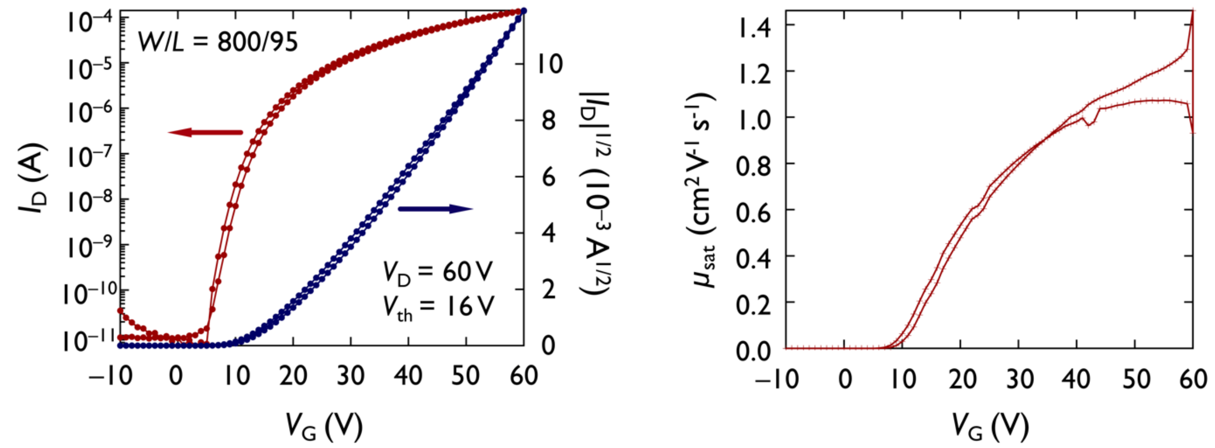

Supplementary Fig. 23 Transfer characteristics and μ_{sat} of 20 nm-thick Cy6–BQQDI polycrystalline OFET on HMDS with a channel length of 100 μm.

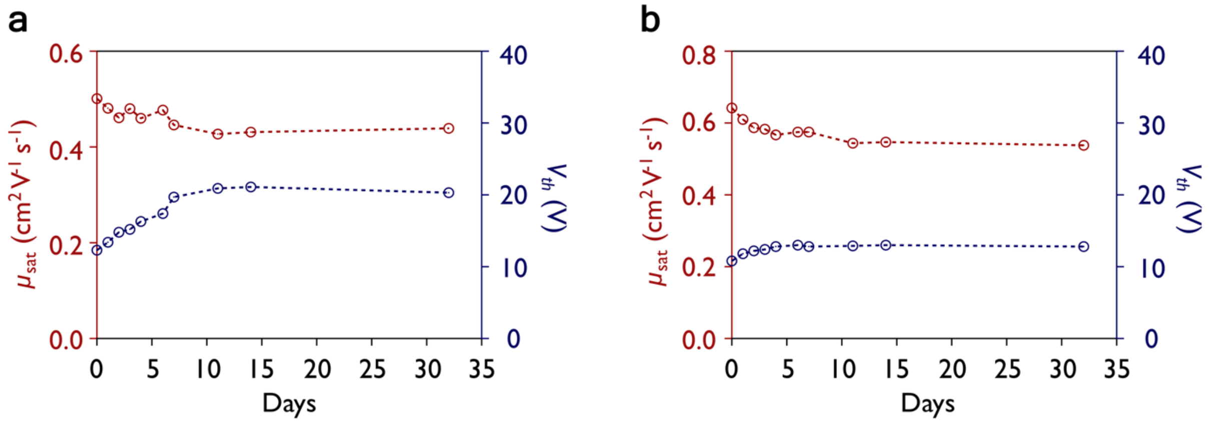

Supplementary Fig. 24 Stability of μ_{sat} and V_{th} of 40 nm-thick Cy6–BQQDI-based polycrystalline OFETs on **a** DTS and **b** HMDS.

11. 2D LUMO Bands

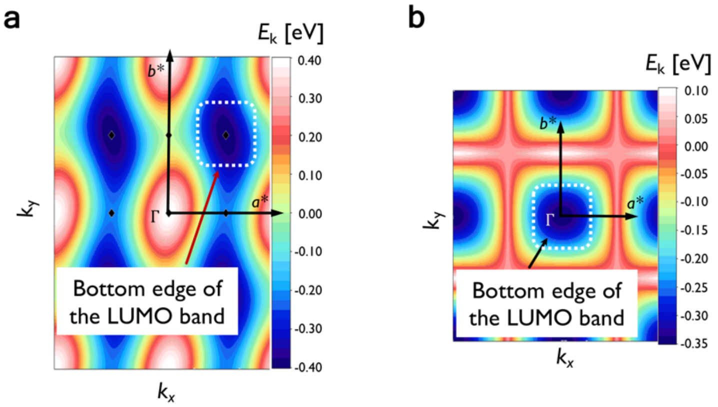

Supplementary Fig. 25 Bottom edges of the 2D LUMO bands for **a** Ph–BQQDI and **b** Cy_6–BQQDI that effective mass values are calculated from.

12. Anisotropy of Electron Mobility

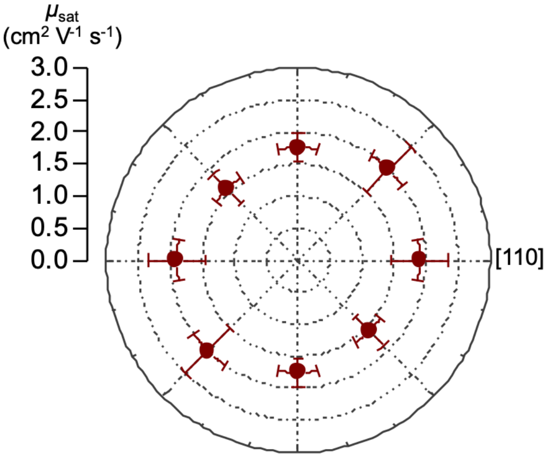

Supplementary Fig. 26 The anisotropy of μ_{sat} of single-crystalline Cy$_6$–BQQDI measured by constructing the OFET channel (channel length = 50 μm) at 0˚, 45˚, –45˚, and 90˚ relative to the crystal growth direction [110], where each data point is extracted from 4–5 devices. Error bars for angle were set to be ±10˚, and errors bars for mobility were given by standard error.

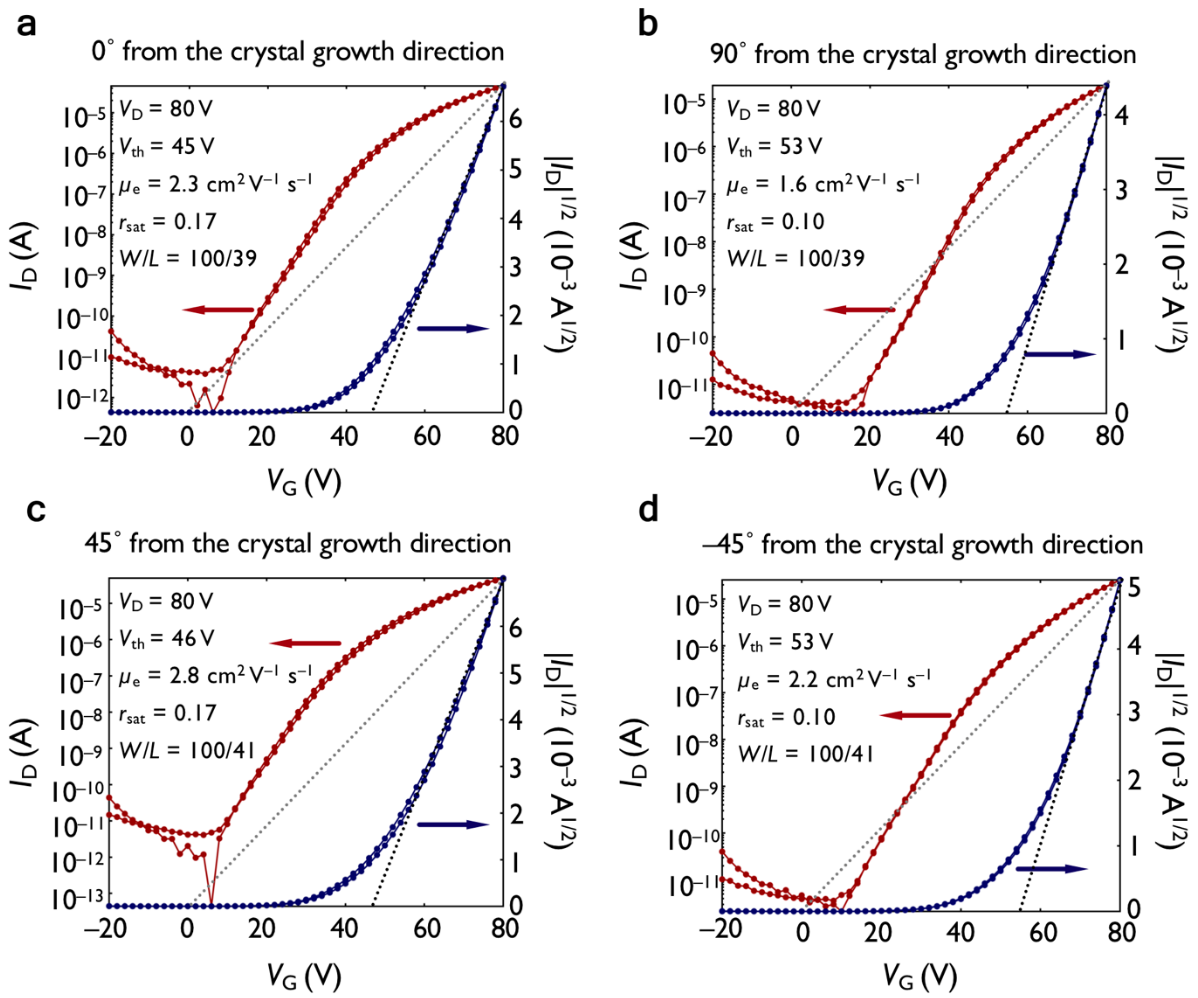

Supplementary Fig. 27 Representative transfer characteristics of single-crystalline OFETs of Cy_6–BQQDI with channels set along **a** 0˚, **b** 90˚, **c** 45˚, and **d** –45˚ from the crystal growth direction [100].

13. Supplementary References

1. Okamoto, T. *et al.* Robust, high-performance n-type organic semiconductors. *Sci. Adv.* **6**, eaaz0632 (2020).
