# Supplementary material for: Approaching isotropic charge transport of n-type organic semiconductors with bulky substituents
Source: Commun Chem. 2021 Nov 11;4:155. doi: 10.1038/s42004-021-00583-2 (PMC9814529; doi:10.1038/s42004-021-00583-2)
Supplement: Supplementary file 8 — Description of Additional Supplementary Files [file 42004_2021_583_MOESM8_ESM.pdf]

Description of Additional Supplementary Files

File Name: Supplementary Data 1

Description: Crystallographic data of Ph-BQQDI

File Name: Supplementary Data 2

Description: Crystallographic data of Cy6-BQQDI

File Name: Supplementary Data 3

Description: MD data of Ph-BQQDI

File Name: Supplementary Data 4

Description: MD data of Cy6-BQQDI (A-form)

File Name: Supplementary Data 5

Description: MD data of Cy6-BQQDI (B-form)
